# Modelling the Lodi, 2023 and Fano 2024, Italy Dengue Outbreaks: The Effects of Control Strategies and Environmental Extremes

**DOI:** 10.1155/tbed/5542740

**Published:** 2025-09-24

**Authors:** Steven M. White, Sandeep Tegar, Bethan V. Purse, Christina A. Cobbold, Dominic P. Brass

**Affiliations:** ^1^UK Centre for Ecology and Hydrology, Benson Lane, Wallingford, Oxfordshire, UK; ^2^School of Mathematics and Statistics, College of Science and Engineering, University of Glasgow, Glasgow, UK

**Keywords:** *Aedes albopictus*, arbovirus, control, dengue, mosquito-borne disease, outbreak, public health

## Abstract

Autochthonous cases of dengue in Europe are increasing. In 2023 (Lodi province) and 2024 (Fano, Pesaro and Urbino province), Italy saw the largest modern dengue outbreaks to date. Public health measures were adopted to mitigate transmission. The efficacy of these measures is unknown. We model the 2023 and 2024 dengue outbreaks to estimate the likely date of introduction of the primary case and efficacy of control measures, exploring explanations for the patterns of dengue cases for the two outbreaks. We apply a climate-driven mathematical model for *Aedes albopictus* and dengue virus transmission to the 2023 and 2024 outbreaks, comparing outputs to case data. The model accurately predicts the initial timeline of the Lodi dengue outbreak (*R*^2^ = 0.83), with a peak in cases in late August 2023, after which the control efforts reduced transmission. The model also accurately predicts the Fano dengue outbreak (*R*^2^ = 0.65), showing an increase in cases, peaking in mid-September 2024, after which there was an abrupt fall in cases. Our results suggest this can be attributed to substantial rainfall, and that public health measures may have latterly prevented a second peak in cases. The high predictive and explanatory ability of the model when applied to the Lodi and Fano outbreaks indicates that this framework may be of high value for public health decision-making for predicting the frequency and magnitude of future dengue outbreaks when coupled with real-time case data.

## 1. Introduction

Dengue virus is the most prevalent global arthropod transmitted virus (arbovirus) and is predominantly vectored to humans by the highly invasive mosquitoes *Aedes aegypti* and *Ae. albopictus* [1]. Human infections range from subclinical infection to dengue fever (flu-like symptoms), dengue haemorrhagic fever, and eventually dengue shock syndrome [2]. 2024 was the worst year on record for dengue incidence with over 10 million cases across 176 countries, with more than 24,000 severe cases and 6508 deaths [3].

In Europe, the number of dengue cases, both imported and autochthonous, are increasing in magnitude [4, 5] and frequency [6]. *Ae. albopictus*, the main European vector of *Aedes*-borne diseases, is now widely distributed throughout Europe [7], and better adapted to temperate regions due to its ability to survive the winters through diapause, an actively induced dormancy state, compared to the principally tropical *Ae. aegypti*. *Aedes*-borne disease outbreaks in Europe currently rely on importation from endemic countries often by viraemic travellers, with dengue being the most frequently imported [4, 5]. These frequent imports have the potential to impact human health ranging in magnitude from sporadic cases to outbreaks [7].

In 2023, a small Italian village of approximately 4500 inhabitants in the Lodi province of the Lombardy Region, reported a large dengue (DENV-1 serotype) outbreak, with an estimated 45 cases [8]. After the diagnosis of the first autochthonous index case on 18^th^ August 2023, public health measures, including epidemiological investigation and vector control measures, were carried out [8]. The identity and timing of the primary case, a likely viraemic traveller from a dengue endemic country, most likely in South America [9], has not been determined. About 2 weeks after the implementation of the public health measures, the number of cases decreased [8].

In the following year (2024), the Italian town of Fano, Marche, experienced the largest known European modern dengue (DENV-2 serotype) outbreak to date [7]. The outbreak was first detected in early September 2024, resulting in over 200 cases (confirmed and probable) by the end of October 2024, peaking in mid-September [10] before falling sharply. The timing of the first imported primary case, a probable viraemic traveller, is also not known [10], making it difficult to predict the lag between a viraemic host arriving and secondary cases to inform mitigation. As the outbreak progressed, Italian officials began an initial mosquito control programme on 11^th^–12^th^ September 2024, using a combination of larvicides, adulticides and removing breeding sites close to the residence of the index case [10], akin to the Lodi control measures. Further treatments were conducted in mid-September and early October. However, throughout the Fano dengue outbreak, there were notable precipitation events that were absent from the Lodi outbreak. The population dynamics of *Ae. albopictus* have a complex relationship with precipitation and temperature [11, 12]. Temperature directly affects traits such as fecundity and survival, whilst precipitation creates breeding sites, whereas extreme precipitation can lead to juvenile mortality via flushing from such breeding sites. Precipitation can also have indirect effects on mosquito population dynamics, for example the lower temperatures associated with precipitation events can reduce the development rates of juveniles and transmission critical adult traits such as the biting rate [11, 12]. To inform future interventions, it is critical to disentangle the role of public health interventions and extreme weather events in producing the sharp drop in cases, which we aim to address here using a predictive model.

Predicting the dynamics of *Ae. albopictus* and dengue transmission for operational use, to support policy and decision-making, remains an open challenge, especially in a One Health framework [13]. In a recent systematic review of predictive dengue outbreak models [14], it was shown that few models were externally validated against data, with some models making inaccurate predictions, thus undermining their usefulness for decision-making. Some statistical models have been used in early-warning systems [15], but they generally are highly specific and are not generalisable for scenario testing. Numerous candidate mechanistic models have been developed, each having drawbacks, depending on their intended use. For example, R0 modelling approaches [16, 17] are extremely useful making quantitative comparisons of transmission risk by different infectious disease across global scales [17, 18], but due to their dynamically decoupled derivations, predicting fine-scale temporal dynamics can come with major caveats. In contrast, fully mechanistic dynamical models [12, 19–21], often systems of dynamical equations, can capture complex processes such as lagged environmental effects on mosquito populations and transmission but require extensive data and high computational power.

In this paper, we test a recently developed state-of-the-art mechanistic dynamical model for predicting dengue outbreaks vectored by *Ae. albopictus* [12], to predict and understand the dynamics of the Lodi 2023 and Fano 2024 dengue outbreaks. We compare model outputs with local health authority case data [8, 10], which reveals information on the control efficacy and explains patterns of dengue cases. This analysis provides an important proof of concept for developing a model for operational public health decision-making.

## 2. Methods

We use a state-of-the-art dengue-*Ae. albopictus* model [12], which we briefly summarise here (Figure S1). The model is a system of environmentally driven stage and phenotypically structured delay-differential equations that represent the temporal transmission of dengue virus between humans by mosquito vectors, predicting mosquito population, trait dynamics and human infections. This model explicitly captures the effects of environmental variation (e.g., temperature, precipitation, evaporation, photoperiod and larval density) on mosquito and virus traits such as mosquito development rates and through-stage survival, mosquito biting rate and the viral extrinsic incubation period (the time taken from taken an infected bloodmeal to a mosquito becoming infectious). We explicitly model the mosquito juvenile stages in dynamic standing water bodies that enable us to incorporate important *Ae. albopictus* behaviours, such as egg diapause and quiescence, larval competition and larval and pupal flushing, a process whereby the body of water overflows and individuals are swept away [22]. The model is currently developed for predicting the dynamics of dengue outbreaks, beginning with the introduction of humans, infected with a single serotype of dengue virus, into a completely susceptible population. The model operates at a 2 km × 2 km resolution.

The model has been extensively validated against independent, global datasets of historic multi-life-stage mosquito abundance and trait timeseries, as well as dengue outbreaks primarily vectored by *Ae. albopictus*. In each validation the model performed with high accuracy, without the need for statistically back-fitting unknown epidemiological parameters [12]. Extrapolating the model spatially by using location specific remotely sensed environmental data and human density estimates, the model pinpointed the high-risk dengue locations throughout Europe, with a high degree of overlap with the locations of historic autochthonous dengue cases [12].

Here, we test the predictive ability of the model by modelling the Lodi 2023 and Fano 2024 dengue outbreaks, which is the first time that the model has been tested beyond the historic training and validation datasets, that included sporadic European outbreaks where the model accurately predicted the total numbers of cases [12]. Case data from the Lodi and Fano outbreaks were selected due to the size of the outbreaks, they were likely to be initiated by single introduction and were attributable to a single serotype in each outbreak, unlike the Rome 2023 outbreak [23]. Although the two outbreaks were caused by different serotypes, DENV-1 and DENV-2, there is no known difference in incubation times between serotypes [24], and therefore, we assume can be modelled equivalently for the purpose of this study. To calibrate the model for the two outbreaks, location specific variables are required over the outbreak timeframe, namely environmental data and human density estimates. Daily temperature, precipitation and evaporation data were obtained from the ERA5-land climate reanalysis dataset accessed from the Copernicus Climate Data Store [https://cds.climate.copernicus.eu/] for the period 18^th^ November 2021–31^st^ December 2023 for Lodi, Italy (45.1300°N, 9.4100°E, WGS84 datum) and 18^th^ November 2021–19^th^ November 2024 for Fano, Italy (43.8200°N, 13.0260°E, WGS84 datum). The environmental data preceding 2023 and 2024 was used to allow burn-in and removal of any transient mosquito dynamics before a viraemic human is introduced to begin the outbreak. Human density over the Fano region was estimated by analysing gridded population data from the 2021 Population and Housing Census, available on the Eurostat web portal [https://ec.europa.eu/eurostat/statistics-explained/index.php?oldid=596753]. The spatial extent of the outbreaks is approximately the resolution of the model (4 km^2^) [8, 10]. To align the resolutions of the model (4 km^2^), environmental data (approximately 100 km^2^) and human population data (1 km^2^), we resampled the environmental data down to the scale of the model grid size by assuming the environment is homogenous across each 4 km^2^ falling within the larger environmental grid cell. For human density, in case Fano, a total of 20 grid cells of 1 km^2^ surrounding the Fano epicentre were analysed. The average population density across these grids was calculated to be 2129 people per km^2^. To estimate the population density at the model resolution, this average was multiplied by four, yielding a population density of 8516 people per 4 km^2^. These steps were taken to counter spatial biases in human population density from rural and peri-urban areas. In contrast, for the village in Lodi, the outbreak epicentre falls entirely within four contiguous 1 km^2^ grid cells, thus matching the model resolution. Hence, we summed across the four grid cells which gave a human population density of 4435 people per 4 km^2^.

Since the introduction dates for the assumed viraemic traveller returning to Lodi and Fano are unknown (the primary cases) [8, 10], we estimate the most likely candidate for the introduction date and error margins around it by optimising the model to the observed case data (all other model parameters are fixed), assuming a single viraemic individual is introduced on a given day, which is varied from 21^st^ May 2023 (Week 20) to 18^th^ June 2023 (Week 24) for Lodi and 3^rd^ July 2024 to 31^st^ July 2024 for Fano to allow a sufficiently large period of time before the probable primary and index cases [8, 10]. Optimisation is achieved numerically by minimising the Kling-Gupta efficiency (KGE) [25], which is a comprehensive metric that rectifies some of the shortcomings of the commonly used metrics, such as RMSE and *R*^2^, taking into account correlation, bias and temporal variability. KGE not only provides a metric on the accuracy of the model, but also on the model ability to reproduce the variability and timing of data, which are important features of temporal epidemic data and are penalised by RMSE and *R*^2^ metrics [25]. Here, the error margins represent the dengue dynamics resulting from introductions 2 weeks before and after the date we identify as the most likely candidate (roughly the generation time—the sum of the extrinsic and intrinsic incubation periods). Introduction date is the only unknown parameter in the model, and all other model parameters are as described by Brass el al. [12]. The model is only compared to the data up until 27^th^ August 2023 for Lodi and 16^th^ September 2024 for Fano so that the effects of the control strategies, which are not explicitly modelled, do not confound the optimisation procedure, but allow case data to be included that are unlikely to be affected by control strategies.

All dengue case data (time of symptom onset) are obtained from published data for Lodi 2023 [8] and Fano 2024 [10], incorporating both probable (consistent dengue infection symptoms with a positive serology for IgM antibodies) and confirmed (detection of viral DNA or antigen, or confirmed antibody neutralisation or antibody increase) cases. We use aggregated weekly case data for the Lodi outbreak to reduce the stochasticity in the data. In Lodi, vector control measures were carried out shortly after the first diagnosed autochthonous index case on 21^st^ August 2023. In Fano, a series of public health measures were implemented from 11^th^ September 2024, after which there is a reported sharp drop in cases. We test alternative explanations for the drop in cases in Fano using the model.

All model code, written in the Julia programming language, is available on GitHub [26].

## 3. Results

**Figure 1:**
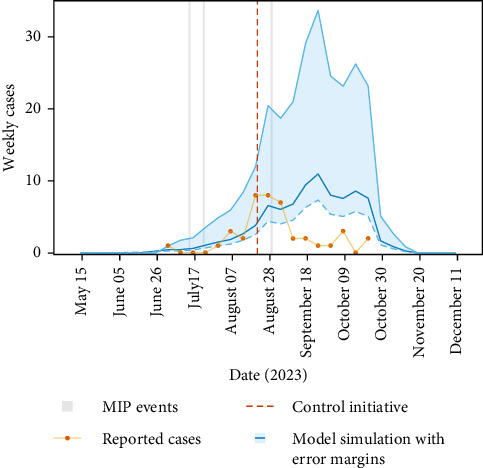
Comparison of model predictions and dengue case data for Lodi, 2023. Weekly newly reported cases (time of symptom onset) over time plotted in orange dots [8]. Model predictions from the dengue model [12] are plotted in blue with the shaded area denoting the error margin after back-fitting the introduction date. The error margins represent the range of introduction dates around the most likely candidate date, with bounds set by deviations from the optimum KGE, defined as dates two weeks before and after the optimum date. The vertical red dashed line denotes the timing that the control measures started in Lodi, and the grey vertical lines denote the days of medium intensity precipitation (MIP) events [27].

For the Lodi, 2023 dengue outbreak, simulating the model and backfitting the introduction date resulted in 4^th^ June 2023 (KGE = 0.68 at minimum, error margin from 21^st^ May 2023 to 18^th^ June 2023) as the most likely candidate date of introduction (Figure 1). The model accurately predicts the rise in cases (*R*^2^ = 0.83 over the optimised period), with an increase in dengue cases up until and beyond the control measure period, unlike the case data which drops sharply approximately 3 weeks after the control measures are used (indicated by the vertical red dashed line). The model predicts that in the absence of control measures, that the dengue cases would have peaked in the latter half of September 2023 before the environment made transmission unsuitable in mid-November 2023. Our analysis suggests that the control measures may have prevented approximately 45 additional autochthonous dengue cases in Lodi. The precipitation event (depicted by the vertical grey line in Figure 1) had a negligible effect on the predicted number of cases (Figure S2).

**Figure 2:**
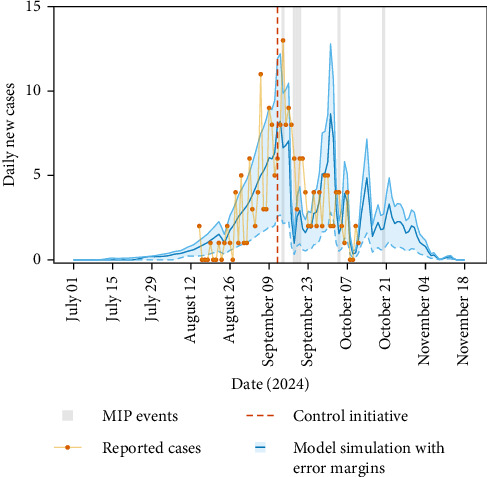
Comparison of model predictions and dengue case data for Fano, 2024. Daily newly reported cases over time plotted in orange dots [10]. Model predictions from the dengue model [12] are plotted in blue. The shaded area denotes the error margins. The error margins represent the range of introduction dates around the most likely candidate date, with bounds set by deviations from the optimum KGE, defined as dates two weeks before and after the optimum date. The vertical red dashed line denotes the timing that the control measures started in Fano, and the grey vertical lines denote the days of medium intensity precipitation (MIP) events [27].

For the Fano, 2024 dengue outbreak, the model suggests an introduction date of 17^th^ July 2024 (KGE = 0.60 at minimum, error margin 3^rd^ July 2024–31^st^ July 2024) as the most likely candidate date of introduction (Figure 2). The model accurately predicts the pattern in cases (*R*^2^ = 0.65 over the fitted period), with an increase in dengue cases up until mid-September, followed by a dramatic decrease in cases, the timing of which occurs shortly after the time at which the control programme began (indicated by the vertical red dashed line). The human incubation period for dengue is approximately 3–10 days [28], and therefore effects of adulticides would not be observed until at least 3 days after application. Similarly, any effects of the larvicide would not be observed after a substantial delay due to the additional larval and pupal development times and the extrinsic incubation period. However, since the model does not include any control mechanisms and that the model continues to be accurate after control begins, we conclude that the dramatic drop is not due to control efforts over this period, unlike the Lodi scenario. However, the model does predict a large second peak in the absence of control at the end of September and early October, which suggests that the control programme may have suppressed this peak.

**Figure 3:**
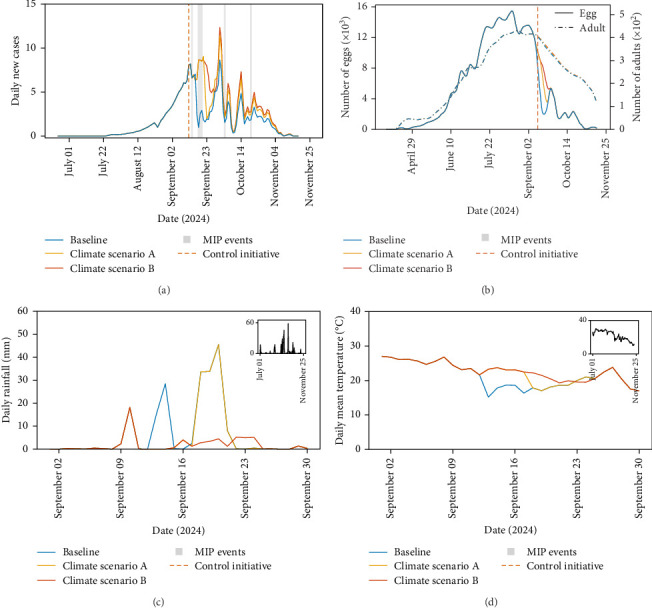
Model output comparisons for three climate scenarios in Fano, 2024. In (A), we plot model new daily dengue case predictions for baseline (blue), climate scenario A (yellow) and climate scenario B (red). Similarly, in (B) we plot the corresponding oviposition and adult abundance for the 3 climate scenarios. In (C, D), we plot the daily rainfall and average daily temperature for the three climate scenarios, respectively. The inlays show the rainfall and temperatures over the outbreak period for the baseline scenario. The new daily dengue cases predicted by the model under each climate scenario are given by the recruitment term into the human infected class as described by Brass et al. [12].

The question remains, if control measures were not responsible for the dramatic decrease in cases in Fano in mid-September, what was? In Figure 2, we highlight a series of medium intensity precipitation events (20–60 mm daily rain [27]), depicted as vertical grey bars. We highlight these since precipitation and the correlated reduction in temperature can affect important *Ae. albopictus* behaviours (e.g., egg diapause and quiescence, larval competition and larval and pupal flushing and adult biting [12, 22, 29]). To understand the effect of the precipitation events we modify the precipitation and temperature data in a systematic way for two different scenarios. In Climate Scenario A, we replace the precipitation event over 13^th^ and 14^th^ September with historic (over 2019–2023) averages of precipitation and associated temperatures. For Climate Scenario B in addition to A, we also replace the subsequent moderate precipitation event, so that the precipitation and temperatures over this period are replaced by historic averages from 13^th^ to 20^th^ September. We depict Climate Scenarios A (yellow line) and B (red line) in Figure 3C,D, where the baseline unaltered climate variables are plotted for comparison (blue line). The model is rerun using these altered climate data, comparing the model outputs to the simulation run with the unaltered baseline climate data (Figure 3A,B).

Figure 3A shows that by comparing the yellow (Scenario A) and blue (Baseline) lines that the dramatic drop in dengue cases in Fano, 2024 would not have occurred if it had not rained heavily, with the predicted number of cases continuing to rise into the latter half of September, which is exacerbated further when we remove the subsequent heavy rainfall event (red line, Scenario B). Therefore, our model suggests that the dramatic fall in case data was predominantly caused by heavy rainfall as opposed to control efforts.

**Figure 4:**
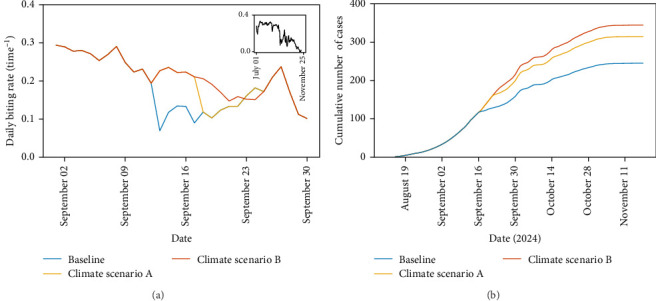
Predicted biting rate (A) and cumulative dengue cases (B) for the 3 climate scenarios for Fano, 2024. The colours depicted correspond to the climate scenarios as shown in Figure 3. The biting rate is defined as the rate at which female mosquitoes take blood meals, which is temperature dependent, and the inverse of the length of the cycle in which adult females mature a new batch of eggs [12].

Why did the extreme weather decrease dengue cases, reducing the peak in transmission in Fano, 2024? The time between the first extreme precipitation event and dramatic fall in dengue cases is too short to attribute to effects on juvenile dynamics as stage-specific developmental lags are too long for extreme precipitation to cause sufficient juvenile mortality by flushing [11, 12]. Indeed, we predict that the larval to adult development time at the time of the control efforts is 10.9 days (95% CI: 9.9–11.8 days). Therefore, the fall in cases is most likely due to instantaneous changes in adult mosquito traits. In our model, it is assumed that adult mosquito traits are not directly affected by precipitation (due to data paucity for accurate mechanistic modelling of the process) but rather are only affected by temperature through changes in adult mosquito mortality and biting rates. In Figure 3B, we plot the changes in adult abundance and oviposition activity over time for the three climate scenarios, which shows little difference in adult abundance, thus indicating no substantial effect of heavy rainfall on adult survival. There is a notable change in oviposition activity for Climate Scenarios A and B, but this has little effect on adult abundance and hence dengue virus transmission. However, mosquito biting rate, the interval between successive adult female blood meals, drops substantially when the rainfall increases and temperature decreases (Figure 4). This in turn decreases dengue virus transmission and hence the drop in reported dengue cases. In conclusion, our model suggests that the lower temperature associated with the heavy precipitation reduced the peak dengue transmission in Fano, 2024, through impacts on the biting rates, which we estimate reduced the number of cases by 27.75% (comparing Baseline and Climate Scenario B, Figure 4B).

## 4. Discussion

The epidemiology of dengue in Europe is rapidly changing. Over the last 20 years the continent has moved from being unsuitable, to isolated and sporadic incidence of autochthonous transmission, to more frequent large scale outbreaks [5–7, 10], with the outbreak in Fano, Italy, 2024, being the largest to date [10]. Therefore, stronger public health strategies are now required to slow down the now unavoidable rise in European dengue cases. Modelling is likely to play an important role in informing such strategies, with model accuracy being an important component of decision-making, affecting credibility [30].

Here, by using a state-of-the art climate-driven dengue model [12], we have been able to rapidly (1) identify the most plausible introduction date of two Italian outbreaks, (2) show that the model can accurately predict the number of expected dengue case using only climate and human population data, and (3) identify the causes of temporal change in dengue cases, disentangling the effects of control measures and climatic effects, and thus contributes to assessment of the efficacy of control practices. We have focussed on two aspects of the model fit in both outbreaks, namely accuracy and trend. Our model explains much of the variance in the data (*R*^2^ = 0.83, Lodi 2023 and *R*^2^ = 0.65, Fano, 2024), but the dengue case data for both outbreaks includes a number of biases and delays in reporting [8, 10], which in turn may also add to stochasticity to the data, thus limiting the model fit.

There are several assumptions within the model that warrant further discussion. For example, our model does not consider aspects such as socio-economic and spatial factors, which are known to be important [31]. Furthermore, the mechanistic modelling framework uses laboratory derived parameters and is coupled to commonly used environmental data [19–21]. Humidity is a known factor in affecting mosquito fitness and arbovirus transmission, but is often overlooked in mechanistic modelling, as we have done here, due to a paucity of quantifiable data to produce the environment-trait functions utilised by mechanistic models [32]. Furthermore, we do not explicitly model how microclimates and human and mosquito behaviour may affect transmission. Despite these omissions, our model broadly predicts the case data (up until control measures are used, and after in the case of Fano).

The model has some limitations in its general utility. Although the assumption of a wholly susceptible human population is suitable for the current European context, where human populations are currently naïve, further model development is required to account for the more complex immunological structures that arise when dengue is endemic and multiple serotypes circulate concurrently. Extending the model to include such immunological detail would expand the model's scope geographically and temporally and increase it's generally utility beyond the current European-like context. Currently, the model is solely parameterised for dengue virus transmission by *Ae. albopictus—*the presence of *Ae. aegypti* is likely to affect transmission, and therefore, the model is unlikely to be suitable in its current format where the two mosquito species co-occur. However, the model is currently being developed to predict the transmission of chikungunya virus, which requires additional information on the environmental sensitivity of the vector competence and extrinsic incubation period in *Ae. albopictus*, which we will report on elsewhere.

Mechanistic modelling approaches have advantages over correlative models that may be less able to cope with population level impacts of extreme climate variability and produce fine grained temporal forecasts [33] needed to inform timing of public health interventions, such as vector control and public information campaigns [13]. Furthermore, this modelling framework could be developed as an operational model for European dengue outbreaks, to analyse real-time dengue data, providing insights and recommendations to help decision-makers at local, regional, national or European levels make informed choices regarding day-to-day operations, with the goal of optimising efficiency, productivity, and resource allocation [13, 34]. However, as demonstrated by the Lodi 2024 outbreak, the weather can have a large effect on the course of the outbreak, impacting the number of cases, but may be difficult to predict over timescales of a few days to a couple of weeks. Therefore, the effect of uncertainty in future climate projections on predictions of future dengue cases must be considered for real-time decision-making. Furthermore, information on the timing of the primary case is valuable in determining the likely size of the outbreak, although gathering case data will help reduce the uncertainty of the cases. The model is currently designed to predict novel dengue outbreaks within 4 km^2^ to model small town outbreaks or clusters within larger cities with minimal readily available information (environmental and human density data). However, if new foci are formed far from the epicentre, then information on urban infrastructure and human mobility may be required [35]. Therefore, predicting outbreaks in large cities may be more challenging, unless the outbreak is localised around the primary case.

An important feature of our model is its fully dynamic structure, in that each component (e.g., mosquito developmental stage and transmission stage) of the model is coupled and interacts with daily environmental variation [19–21], as opposed to R0 model approaches where the stage-specific environment-trait relationships are decoupled across the mosquito developmental stages [16]. The inclusion of dynamic interactions in our model allows for the subtle effects of environmental extremes to be understood. Due to this dynamic approach and the dengue case data provided [8, 10], we were able to show that the control strategies had a substantial effect on reducing further cases in the Lodi, 2023 dengue outbreak, and that rainfall had a substantial effect of reducing the peak dengue cases in Fano, 2024, although control had a likely effect in preventing a second peak. This was achieved without the need to explicitly model control dynamics. However, if the effect of control strategies at the peak of the Fano, 2024 dengue outbreak, had been stronger, possibly through earlier deployment to reduce peak mosquito biting, then substantial model development would have been required in order to determine the cause of any changes in dengue cases. For control strategies such as the sterile insect technique (SIT) [36], proof-of-concept models have been developed [37]. However, even in this case, to the best of our knowledge, no mechanistic model has been developed that can accurately predict the dynamics of changes in *Ae. albopictus* abundance and subsequent dengue cases after SIT releases. Thus, accurate models of control dynamics remain an open challenge and another important step for an operational decision-support tool. Nonetheless, our framework provides and important basis for accurate models of control dynamics due to the integration of density-dependent processes [12]. Although data on the effects mosquito abundance from control strategies are often available (e.g., [36]), these do not coincide with arbovirus case data in the same location and time. Therefore, extensive validation of independent datasets from multiple outbreaks and control programmes are required to reduce uncertainty in control models.

In this paper, we have suggested that the most plausible introduction of the primary case for the Lodi, 2023 dengue outbreak was 4^th^ June 2023, and for the Fano 2024 dengue outbreak, it was 17^th^ July 2024. These previously unknown primary cases fall within the timeframe of other *Aedes*-borne disease outbreaks in Europe [6]. Therefore, our analysis suggests a substantial time lag between the likely primary and index case dates [8, 10]. Furthermore, the primary case dates fall into the Italian school holiday period, which has been shown, more generally, to correlate with an increase in a wide range of imported infectious disease cases due to increased overseas travel [6, 38]. However, in France, it has been shown that introduction events are highly specific, whereby the introduction sources to France are mainly due to arriving travellers from a small number of countries. For example, in 2024 the French overseas territories of Martinique and Guadeloupe accounted for 54.5% of imported cases [5], despite larger numbers of concurrent cases in South American countries [3]. Therefore, there is a clear need for a better understanding of country-specific importation [39] and travel [40] and how they might affect corresponding dengue outbreaks and be mitigated against.

The effects of climate change, although not considered here, are readily predictable since the relationship between environment and mosquito and virus traits have already been modelled [12]. Coupling the dynamical model with climate model outputs will lead to future disease risk predictions [19], which we have produced for other disease systems using similar modelling techniques [41, 42]. For *Ae. albopictus*-borne diseases, one mechanism of climate change risk is from adult mosquitoes now being able to overwinter and reproduce in Southern Europe [43], meaning that arbovirus outbreaks may not be confined to a single year. Hence, as climate change lengthens the mosquito biting season, the risk of dengue and chikungunya becoming endemic in Southern Europe becomes more likely [44], and making public health decisions in this novel climatic scenario will require modelling support.

The model output shows good agreement with the known case data for the two outbreaks. However, there is likely to be substantial under reporting. Further, the number of unreported cases is likely to have changed over time due to changes in reporting, public awareness and increases in mosquito biting activity. As Sacco et al. [10] show, the reporting time (the time between symptom onset and notification to local health authorities) shortened over the course of the Fano, 2024 outbreak. These dynamic changes in dengue case number report times are likely to have some effects on our conclusions, such as the estimation of the primary case date. Unfortunately, incorporating these dynamics into the model without further data (or making crude hypothetical assumptions) hinders our ability to unravel these effects and remains an open challenge. This highlights the need for timely information on dengue case data and mosquito surveillance, perhaps combining conventional monitoring with novel techniques, such as wastewater-based surveillance [45].

Timely dengue case information flow between local authorities is required for informing operational models so that decisions can be made quickly [13, 14]. Predicting in real-time is important for decision-making, especially when cases are low, as authorities need guidance as to whether those low number of cases will grow into a substantial outbreak or not [13, 30, 34]. This work serves a vital step in providing an accurate decision-support tool, given the evidence of the model's accuracy of predicting European dengue outbreaks. By public health bodies providing near real-time information on case data (both time and location), especially primary cases, the model can be used to make projections on future cases can be quickly calculated and disseminated. Predicted outputs such as the total number of cases and the peak transmission times are likely to help inform decision-makers as to the scale of mitigation measures required and the expected public health burden between varying outbreak locations. Thus, such timely predictions will likely aid vector surveillance and control campaigns, and healthcare preparedness.

## 5. Conclusion

European dengue outbreaks are increasing in frequency and magnitude, and therefore public health risks and decision-making processes are changing accordingly. Using a state-of-the-art model, we were able to rapidly predict and explain the pattern of the Lodi, 2023 and Fano, 2024 dengue outbreaks, the largest modern outbreaks that Europe has experienced to date. Our analysis suggests that the control measures employed in Lodi had a substantial effect in reducing further dengue cases, but in Fano they had a more limited effect in reducing dengue transmission, whereby substantial rainfall events also had an effect in reducing cases. The modelling work is an important step for developing an operational model to predict future outbreaks to help inform policy and decision-making for public health officials. Further framing and testing model developments with public health stakeholders across contexts will help to ensure their value for dengue management.

## Data Availability

The data that support the findings of this study are openly available in sandeeptegar/Fano_dengue_outbreak_2024: Release v1.00.0-Code at https://zenodo.org/records/15267652.

## Supporting Information

Supporting Information The file contains a model overview S1, including Figure S1, a schematic of the stage and phenotypically structured epidemiological model. The file contains addition analysis of the Lodi dengue outbreak showing S2, that the precipitation event had little effect on the number of dengue cases (Figure S2).
