# Supplementary material for: Modelling the Lodi, 2023 and Fano 2024, Italy Dengue Outbreaks: The Effects of Control Strategies and Environmental Extremes
Source: Transbound Emerg Dis. 2025 Sep 24;2025:5542740. doi: 10.1155/tbed/5542740 (PMC12488316; doi:10.1155/tbed/5542740)
Supplement: Supporting Information — The file contains a model overview S1, including Figure S1, a schematic of the stage and phenotypically structured epidemiological model. The file contains addition analysis of the Lodi dengue outbreak showing S2, that the precipitation event had little effect on the number of dengue cases (Figure S2). [file 5542740.f1.docx]

**Supplementary Material: Modelling the Lodi, 2023 and Fano 2024, Italy dengue outbreaks: The effects of control strategies and environmental extremes**

**S1. Model Overview**

The model framework used to predict the dengue outbreaks in Lodi 2023 and Fano 2024 is described by Brass and collagues [1]. Here, we give a brief overview of the model.

To model the trait and population dynamics of *Aedes albopictus*, the model uses a continuous time delay differential equation approach [2] with an additional phenotypic structure to represent the effects of within and between generation plasticity [3]. This approach is flexible and able to incorporate the effect, both instantaneous and delayed, of environmental stressors on multiple traits, making it ideal for accounting for the complex life history of *Ae. albopictus*. Following Brass and colleagues [3], for a generic stage-structured population, $N$, the density of individuals in life stage $i$ and phenotypic class $j$ at time $t$ is described by a system of equations given by

$\frac{dN_{i,j}\left( t \right)}{dt}=R_{i,j}\left( t \right)-M_{i,j}\left( t \right)-D_{i,j}\left( t \right)$,

for $i\in1,\cdots,n$ and $j\in1,\cdots,m$. Here, $R_{i,j}\left( t \right)$, $M_{i,j}\left( t \right)$ and $D_{i,j}\left( t \right)$ are functional forms describing the recruitment, maturation and death rate of individuals in life stage $i$ and phenotypic class $j$, respectively. These are often dependent on biotic and abiotic environments, parameterised by experimentally derived reaction norms describing the environment-trait relationship [1, 3].

The *Ae. albopictus* model predicts the dynamics of a population of mosquitoes arising from a single water body of fixed dimensions (Fig. S1). The model inputs are environmental variables (temperature, precipitation, evaporation and photoperiod) from the location being simulated and the outputs are predictions of population and trait dynamics (see Methods). Adult mosquitoes oviposit eggs either onto the surface of the water or around the sides of the habitat, with the proportion of eggs being placed around the side of the habitat increasing as water level decreases. The eggs placed into or around the water body express either a diapausing ($E_{D}$) or non-diapausing ($E_{\gamma}$) phenotype which is determined by a maternal effect in response to falling temperatures and decreasing photoperiod. Once development is complete, both diapausing and non-diapausing eggs either become quiescent ($E_{Q}$) to survive dry periods or immediately hatch into larvae ($L$). Quiescence continues until the dormant egg is inundated by precipitation after which it immediately hatches. The development and survival of eggs are assumed to be temperature dependent, with diapausing eggs also being photoperiod dependent. The water body only varies in response to changes in temperature, the accumulation of precipitation, and through evaporation of standing water and is otherwise identical in every respect between locations.

Larval mosquitoes ($L$) compete for available resources in the aquatic habitat, consisting of a single larval class. Available resource is assumed to be consumed in its entirety and to be replenished daily, representing the product of temperature-dependent metabolic processes in the larval environment. Once larval development is complete pupation begins with the development of pupae (implicitly modelled due to the lack of density-dependence). The container habitats are vulnerable to flushing, a process whereby the body of water overflows and individuals are swept away. Flushing is modelled by increasing the mortality of larvae and pupae whenever the height of the water in the habitat exceeds the height of the container and rainfall is sufficiently intense. Further, the containers are also susceptible to drying out, and whenever all water within the container evaporates, all non-quiescent juveniles are assumed to die out.

Adult mosquitoes ($A$) experience developmental plasticity in response to their experience of temperature and intraspecific competition as larvae. Each individual’s experience of the average temperature and the average food available per larvae per day over the course of the larval period is used to predict that individual’s wing length. We discretise adults into a large number of coexisting sub-classes, with each adult class determined by their wing length ($A_{i}, i=1,\cdots,m$), thus defining the adult phenotypic structure. Wing length is then used to determine the fecundity and longevity of that individual as an adult. Both fecundity and longevity are then further modified by the current temperature meaning that within the model adult traits respond to both current and historic environmental conditions. The production of cold resistant diapausing eggs is triggered when falling temperatures and photoperiod reach a critical threshold.

The model for the population and trait dynamics of *Ae. albopictus* is incorporated into a compartmental susceptible-exposed-infected-resistant (SEIR) model for dengue virus vectored by *Ae. albopictus* (Fig. S1). The human population is partitioned into those susceptible to infection ($H_{S}$), those infected ($H_{I}$), and those resistant to infection due to having recovered ($H_{R}$) – the exposed class is implicitly modelled. The size of the human population is constant, where the population density is estimated as described in the Methods.

Mosquitoes are assumed to bite at a temperature-dependent rate that is inversely proportional to the length of the gonotrophic cycle. The proportion of uninfected mosquitoes of a given wing length ($A_{i}$) that become infected ($I_{i}$) after biting an infected human ($H_{I}$) is temperature dependent. After a temperature-dependent extrinsic incubation period, an infected mosquito can bite and transmit the dengue virus to an uninfected human ($H_{S}$). After the intrinsic incubation period, the infected human can transmit the infection to new mosquitoes and recovers from the infection after a recovery period.

The specific form of delayed differential equations, along with the environmentally-dependent parameters are fully described by Brass and colleagues [1].


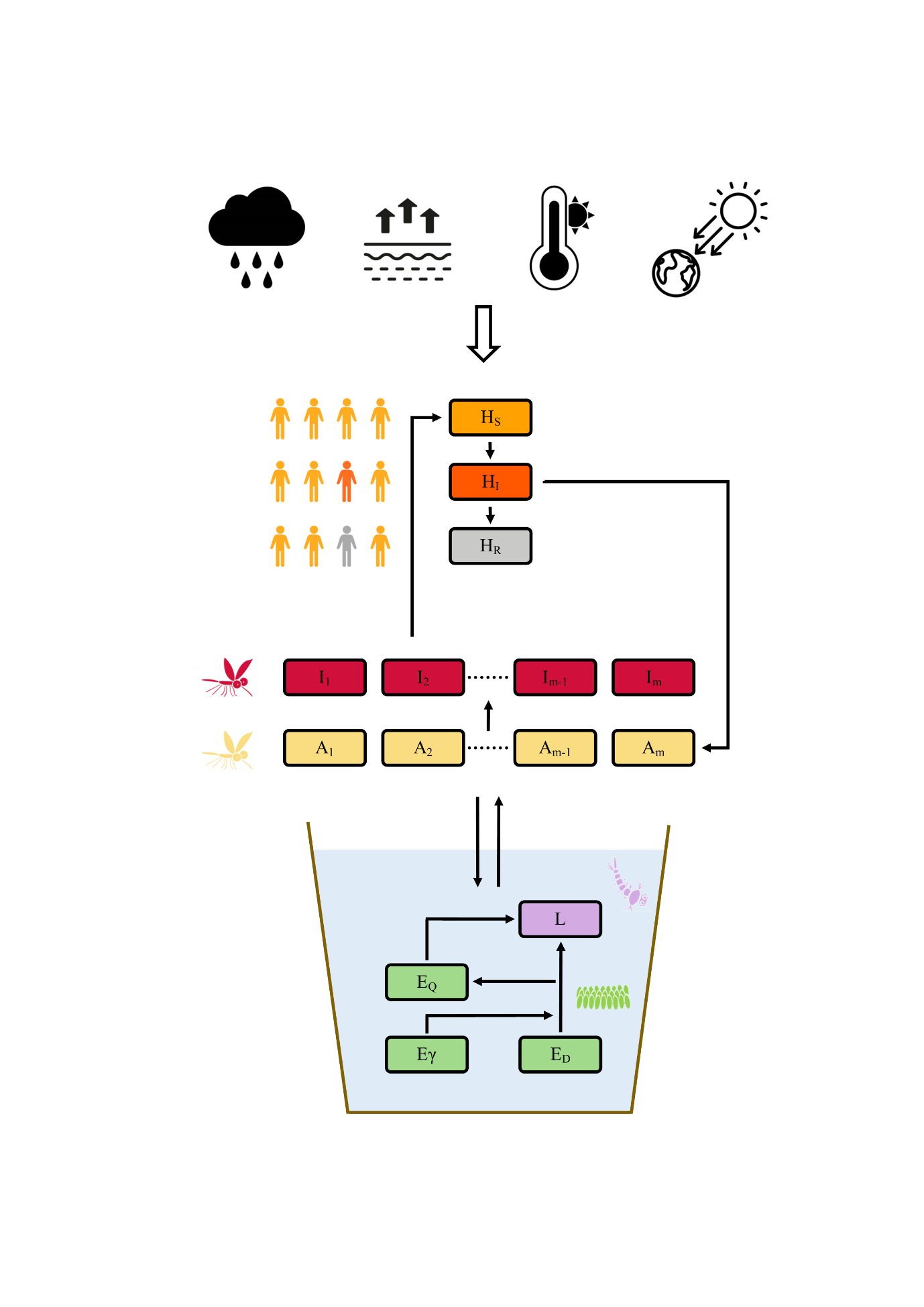


Fig. S1: A schematic of the stage and phenotypically structured epidemiological model. Descriptions of the state variables in the coloured boxes are described in the text.

**S2. The Lodi Dengue Outbreak for the Average Climate Scenario**

To understand the effect of the precipitation event in Lodi 2023 after the control measures were applied (vertical red dashed line, Fig. 2A), we modify the precipitation and temperature data. In the Average Climate Scenario, we replace the precipitation event over 29thth and 30th August 2023 with historic (over 2018-2022) averages of precipitation and associated temperatures. We depict the Average Climate Scenarios (yellow line) in Fig. 2C, D, where the baseline unaltered climate variables are plotted for comparison (blue line). The model is then rerun using these altered climate data, comparing the model outputs to the simulation run with the unaltered Baseline climate data (Fig. S2A, B).


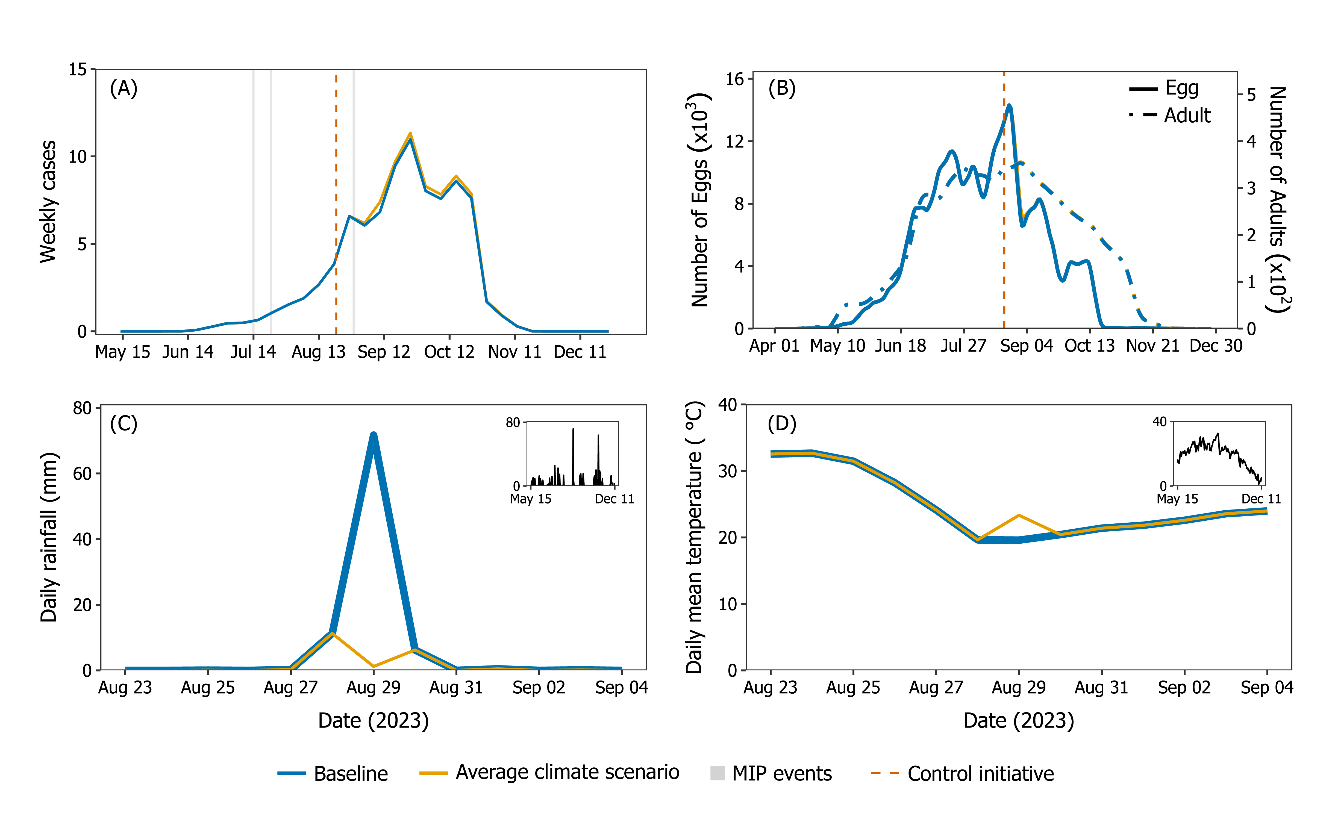


Fig. S2: Model output comparisons for 2 climate scenarios in Lodi, 2023. In A, we plot model new daily dengue case predictions for Baseline (blue) and a modified climate scenario (yellow). Similarly, in B we plot the corresponding oviposition and adult abundance for the 2 climate scenarios. In C and D, we plot the daily rainfall and average daily temperature for the 2 climate scenarios, respectively. The inlays show the rainfall and temperatures over the outbreak period for the Baseline scenario. The new daily dengue cases predicted by the model under each climate scenario are given by the recruitment term into the human infected class as described by Brass and colleagues [1].

Fig. S2 suggests that the difference in the number of cases for the Baseline and Average Climate Scenario is almost negligible, as is the numbers of mosquito eggs and adults. We therefore conclude that the precipitation event after the control measures were applied had little effect on reducing dengue case numbers and that the control measures mainly accounted for the predicted drop in dengue cases in Lodi 2023 [4].

**References**

1. Brass, D.P., et al., *Role of vector phenotypic plasticity in disease transmission as illustrated by the spread of dengue virus by Aedes albopictus.* Nature Communications, 2024. **15**(1): p. 7823.

2. Nisbet, R.M. and W.S.C. Gurney, *The systematic formulation of population models for insects with dynamically varying instar duration.* Theoretical Population Biology, 1983. **23**(1): p. 114-135.

3. Brass, D.P., et al., *Phenotypic plasticity as a cause and consequence of population dynamics.* Ecology Letters, 2021. **24**(11): p. 2406-2417.

4. Rovida, F., et al., *The 2023 dengue outbreak in Lombardy, Italy: A one-health perspective.* Travel Medicine and Infectious Disease, 2025. **64**: p. 102795.
